# Cancer surviving patients' rehabilitation – understanding failure through application of theoretical perspectives from Habermas

**DOI:** 10.1186/1472-6963-8-122

**Published:** 2008-06-06

**Authors:** Thorbjørn H Mikkelsen, Jens Soendergaard, Anders B Jensen, Frede Olesen

**Affiliations:** 1 The Research Unit for General Practice, University of Aarhus, Vennelyst Boulevard 6, DK-8000 Aarhus C, Denmark; 2 The Research Unit for General Practice, University of Southern Denmark, J.B. Winsløws Vej 9, DK-5000 Odense C, Denmark; 3 Department of Oncology, Aarhus University Hospital, Nørrebrogade 44, DK-8000 Aarhus C, Denmark

## Abstract

This study aims to analyze whether the rehabilitation of cancer surviving patients (CSPs) can be better organized. The data for this paper consists of focus group interviews (FGIs) with CSPs, general practitioners (GPs) and hospital physicians. The analysis draws on the theoretical framework of Jürgen Habermas, utilizing his notions of 'the system and the life world' and 'communicative and strategic action'. In Habermas' terminology, the social security system and the healthcare system are subsystems that belong to what he calls the 'system', where actions are based on strategic actions activated by the means of media such as money and power which provide the basis for other actors' actions. The social life, on the other hand, in Habermas' terminology, belongs to what he calls the 'life world', where communicative action is based on consensual coordination among individuals. Our material suggests that, within the hospital world, the strategic actions related to diagnosis, treatment and cure in the biomedical discourse dominate. They function as inclusion/exclusion criteria for further treatment. However, the GPs appear to accept the CSPs' previous cancer diagnosis as a precondition sufficient for providing assistance. Although the GPs use the biomedical discourse and often give biomedical examples to exemplify rehabilitation needs, they find psychosocial aspects, so-called lifeworld aspects, to be an important component of their job when helping CSPs. In this way, they appear more open to communicative action in relation to the CSPs' lifeworld than do the hospital physicians.

Our data also suggests that the CSPs' lifeworld can be partly colonized by the system during hospitalization, making it difficult for CSPs when they are discharged at the end of treatment. This situation seems to be crucial to our understanding of why CSPs often feel left in limbo after discharge. We conclude that the distinction between the system and the lifeworld and the implications of a possible colonization during hospitalization offers an important theoretical framework for determining and addressing different types of rehabilitation needs.

## Background

The number of cancer surviving patients (CSPs) continues to rise, and many have rehabilitation needs [1-3]. Cancer rehabilitation is a complex issue that covers both biomedical and psychosocial aspects [3-5]. The World Health Organization (WHO) describes the scope of rehabilitation as:

"*a process aimed at enabling persons with disabilities to reach and maintain their optimal physical, sensory, intellectual, psychiatric and/or social functional levels...*" [6]

Many different professionals contribute to cancer rehabilitation, including hospital physicians and GPs. [4]. Since January 2007, the municipalities in Denmark have been responsible for providing rehabilitation services and for financing sick leave and pensions. The CSPs' employers are also relevant in regard to maintaining work life, network and living standards [7,8].

Successful rehabilitation requires efficient organization; but in most countries, including Denmark, rehabilitation is not systematically organized. [4,9] This paper aims to analyze how the organization of cancer rehabilitation could be improved and to identify barriers for such improvement. We draw on data obtained by means of focus group interviews (FGIs) with hospital physicians, CSPs and general practitioners (GPs).

### Theoretical framework

We used Jürgen Habermas' "Theory of communicative action" [10] as our theoretical framework. Firstly, Habermas' use of binary codes in the system seems to be able to provide some of the structural reasons for some CSPs' feeling of being left in limbo after discharge. The binary codes work as inclusion/exclusion criteria for, e.g., medical aid, which presupposes an illness, whereas healthy people are not included for treatment.

Secondly, Habermas' theory on colonization of the lifeworld by the system seems able to explain why CSPs often prefer that the rehabilitation is conducted by the hospital staff rather than by professionals in the primary care sector or other settings.

In Habermas' terminology, the social security system and the healthcare system, including actors like hospitals, GPs and primary healthcare, are subsystems belonging to what he calls the 'system'. The social life, on the other hand, in Habermas' terminology, belongs to what he calls the 'lifeworld'.

*The lifeworld is characterized by communicative action. Communicative action refers to interaction that is mediated through talk and oriented to an agreement that will provide a basis for a 'consensual coordination of individually planned plans of action'*[11]

*"Lifeworld represents the everyday social world within which individuals interact with others to decide and organise their affairs in the private sphere of their own families or households or in the wider public sphere. 'The system' for Habermas comprises economy and state, each characterized by strategic action via their respective steering media of money (leading to commodification) and power (leading to juridification or bureaucratisation). When economy and state intrude in inappropriate and unaccountable ways into the lifeworld they can be said to 'colonize' it. In just this fashion the 'voice of medicine' has partially colonized the 'voice of the lifeworld' (Mishler, 1984)." *[11,12]

A central part of Habermas' theory is that the lifeworld can only be reproduced in and by the means of the lifeworld: the common understanding based on what he calls 'communicative action'. [10]

When the system world intrudes in an inappropriate and unaccountable way into the lifeworld the problem is that steering media overrule and to some extent replace the communicative action. This colonization can lead to psychological and social problems such as identity loss and a reduced feeling of social belonging [10].

In practice, we use both worlds, shifting back and forth simultaneously, e.g., it is part of the lifeworld when at the grocery store we meet and talk to a friend about how things are going; but, when a minute later, we are paying for the groceries, we engage in a strategic action that belongs to the system part. Both parts are important reproductive aspects of modern society. While communication aims at mutual understanding, strategic action aims at success, e.g., getting groceries, or within the biomedical discourse: diagnosis or cure.

When patients seek medical advice to ease a problem facing them in their lifeworld, e.g., pain or cough, the problem has to be recognized as a biomedical problem by the physician before further investigation or treatment is instigated. In other words, the patient's symptoms have to fit into the biomedical discourse for the patient to receive medical treatment.

**Figure 1:**
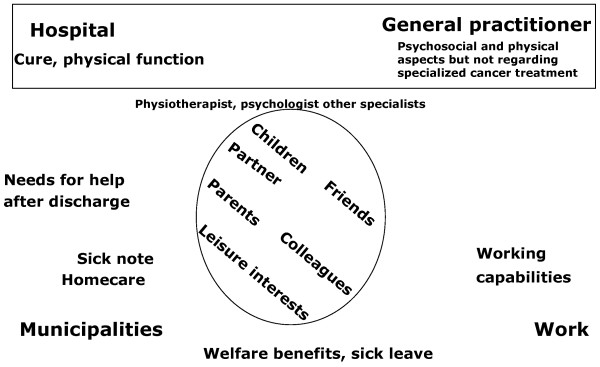
**The circle represents the CSP's lifeworld which includes important actors, such as the CSP's peers.** The remainder of the figure, surrounding the circle, represents the system, which can support the CSP during treatment and rehabilitation. The box surrounding hospital and the GP symbolizes the biomedical discourse.

The central actors in cancer rehabilitation are illustrated in Figure 1: The circle illustrates the CSP and the most relevant peers and actors in the CSP's life. These aspects are described from the CSP's point of view in another paper identifying rehabilitation needs in relation support to the family, social support and relations to friends and acquaintances. That paper also discusses aspects such as psychological help also in relation to fear of relapse as well as the need for continuous support and information about rehabilitation opportunities[5]. The remainder of the figure around the circle illustrates the "system", comprised by the public actors (sub-systems) who can help the patient survive and who can help the CSP in their rehabilitation process. These public actors can refer to each other for additional help or support on different subjects, hopefully enabling the CSP to reach and maintain an optimal level of function. The roles of the GP, the hospital physicians and the municipality are described below.

By applying Habermas, we illustrate that the public sector (the system) aims at supporting the CSP's lifeworld. However, we also introduce the distinction between the CSP's experiences and interactions in the lifeworld vs. the social security system and the healthcare sector which are two different things that work through different means. This duality is important in Habermas' theory since he states that the system can colonize the lifeworld and distort its reproduction.

## Methods

### Sampling

In order to grasp the diversity among the hospitals, we conducted the FGIs among physicians in three types of hospitals: two local hospitals (Herning and Hostebro), a major regional hospital (Vejle) and a university hospital (Aarhus). Similarly, we invited GPs and CSPs working and living in the catchment areas of these hospitals to participate in the FGIs. The FGIs were based on judgement sampling that aimed at maximum variety as we supposed that it could work as a proxy for differences in rehabilitation offers and needs [13]. GPs were sampled with a view to obtaining variation in gender, age, practice type (solo/multi) and urban or rural location. GPs living in the catchment area of Vejle Hospital were members of the county's cancer group and this group of GPs was not asked to identify patients since the sampling strategy for patients was subsequently changed. Interviews with the four GPs in the catchment area of Herning and Holstebro were conducted as individual interviews. The hospital physicians were sampled with the aim of representing different specialities. The CSP FGIs were also based on judgement sampling aimed at obtaining maximum variety in gender, age, cancer type and living in a rural vs. urban area. The CSP characteristics are presented elsewhere [5]. Each GP and hospital physician was asked to identify two CSPs each, which we could invite to participate. The inclusion criteria were that the CSPs should be supposedly free of cancer, between 18 and 75 years of age and able to speak up in a group. The physicians received a description of the project to hand out to the CSPs and asked the CSPs if they would participate. Subsequently we received information from the physicians on the CSPs willing to participate, including information on cancer type, age, name and address. Among the CSPs who were willing to participate, we invited informants according to the judgement sampling described above. CSPs were invited by mail describing the purpose of the study, their participation as well as underlining that if they changed their mind, they could withdraw from the study at any time and that it would not influence their present or future treatments. Subsequently, the CSPs were contacted by phone regarding their continued willingness to participate and to check if they could participate in the FGI according to a pre-established list of possible dates. The study was performed in accordance with the Declaration of Helsinki.

### The interview and processing of data

All interviews were conducted in undisturbed meeting facilities. The FGIs with hospital physicians, GPs and CSPs within the catchment area of Aarhus University Hospital were conducted in meeting facilities at the Research Unit for General Practice in Aarhus.

The remaining two FGIs with CSPs were conducted in meeting facilities at local restaurants, while the other FGIs with hospital physicians were conducted in local meeting facilities.

The interviews were semi-structured, and were conducted according to the GP interview guide in Table 1. Alterations in relation to the FGI guide for hospitals physicians are marked in italics. The interview guide used in the CSP FGIs is presented elsewhere [5]. We used open-ended prompt questions [14,15], based on our research question and extensive literature search.

**Table 1:** FGI guide for GPs. The same questions were used in the FGIs with hospital physicians, with the exception of those written in italics.

Presentation of the project by the means of a poster – the focus area is general practice. (speak loudly and clearly, not at the same time, and be aware of mutual confidentiality)

Opening question:
- A short round where you present yourself by name, age, working place, and type of practice.

- How do you define the concept of rehabilitation for cancer patients?

- How do you see the GP's role in the rehabilitation process? (What and how?)

- What happens when a cancer patient is discharged from the hospital after the end of treatment? (Does the patient contact you? What are the problems? What happens next?)

- What rehabilitation needs do you experience a typical cancer patient to have?

- We are working with a broad definition of rehabilitation. Therefore, I will now ask more carefully regarding specific areas that we would like to cover. Some of the issues have already been mentioned, but we would like to know more about:
- Physical (are the needs met?)
- Psychological
- Social
- Educational and occupational
- Material needs (rehabilitation aids, wage decreases)

- *How do you coordinate and support the patient in the rehabilitation process? (Who can help or assist you? What do you wish for/need? What do you do yourselves?)*

- What rehabilitation possibilities can you offer the patient? (And what is the purpose?)

- What rehabilitation possibilities are needed? (Are they applied?) And who should do the follow-up?

- In the context of the current discussion: What du you think the GP's role in the rehabilitation process is? (Who should coordinate, how should it be prioritized?)

*Danish law says that a plan for effective and quick retraining should be available at discharge. Do you experience that the patients receive this? Does it work as intended?*

- Concluding questions:
- We have discussed rehabilitation, is there anything we have missed?
- Are there any special messages we should bring with us?

Thank you very much

During the interviews, we explored the informants' experiences and attitudes by additional statements, such as "Tell me more about that", but, often, other informants in the group explored the statements and experiences or questioned the perceptions. When the subject of the discussions changed, we conducted an immediate first validation by summarizing the contents of the discussion and asking for further comments.

After each FGI, the interviewers discussed the immediate impressions and results from the FGI, evaluated it and gave each other feedback on the process. THM analyzed the interviews. The interviews were transcribed by one of our two secretaries trained and instructed in transcribing FGIs. The FGIs were transcribed in full, read, and analysed by THM, JS and FO independently. Any differences were discussed until agreement was reached. We applied an inductive approach, focusing on the informants' perceptions, understandings and ideas. We also applied a deductive analytic strategy based on the themes of the interview guide and a pre-established list of CSPs' rehabilitation needs based on the literature (Table 2). The discussions within these groups were analysed phenomenally, focusing on the informants' experience and perception of things and events [15]. CSPs' rehabilitation needs are related to and affect the CSPs' lifeworlds. However, this fact is not displayed in the the health care system's efforts. Our analysis therefore focused on the rehabilitation needs; in particular we investigated how, why and by whom the rehabilitation needs could be met. The study draws on Habermas' theoretical frame work which enables us to analytically distinguish lifeworld from system world aspects. The software NVivo 2.0 was used in the analysis for coding, sorting and retrieving the data.

**Table 2:** Possible rehabilitation needs were the CSP may need information and help

**The physical level**

- Problems stemming form disease and treatment
- Pain
- Amputation
- Late effects stemming from treatment and the disease.
- Problems in understanding what happens
- Symptoms
- Symptom relief.
- Disease/treatments influence on sexual life/body identity
- Nutrition and food
- Life style changes

**The psychological level**

- Crisis
- Chock
- Fear, Fear of death, fear of living,
- Fear of relapse
- Handling of a possible incriminating situation
- Coping strategies.
- Changed identity/identity problems
- Difficulties in letting go of the role of the sick
- Changed quality of life
- Opportunity to meet other patients in the same situation
- Increased risk of depression
- Information on possible genetic disposition
- Information and support enabling the patient to take control and act.

**The social level**

- Body identity
- Sexuality
- Life plans
- Stigmatization
- Accept of being a CSP
- Fear and sorrow among relatives
- Normalization of family and network
- Fear of what is going to happen in the future

- Changes in roles/Status in
- Marriage/relationship
- Family
- Children
- Risk of being childless
- Single parents
- Risk of loosing a breadwinner
- Workplace
- Friends
- Leisure activities (e.g. sports)

- Self-help groups/patient organisations for patient and relatives

**The vocational level**

- Keeping contact to workplace
- Working on reduced hours
- Unemployment (fear of)
- Reduced working capacity
- Sick note
- Vocational rehabilitation
- Early retirement/retirement

**The material level**

- Reduced income
- Expenses in relation to aids and appliances,
- Problems in relation to pension (now or for sawing)
- Material comforts and/or reduced living standard.
- Economic problems
- Reduced living standards (reduced housing standard due to reduced income)
- Problems regarding information on rights
- Problems in relation to social and economic conditions
- Problems in relation life plans

## Results

The results section focuses, firstly, on the role of GPs and hospital physicians in cancer rehabilitation. This is followed by a section on actual rehabilitation offers and the knowledge of the CSPs' rehabilitation needs. Finally, we analyze the organization of cancer rehabilitation and how it works.

### The different foci of GPs vs. hospital physicians concerning rehabilitation

Physical and psychosocial aspects are different aspects of cancer rehabilitation. In our interviews, this difference is apparent in the different foci of hospital physicians and GPs. The hospital physicians focus primarily on the physical aspects of rehabilitation:

Hospital physician: "*We need to know if the artificial limb works, that's our primary interest.*"

Hospital physicians do not think that the hospital has the resources to take proper care of the psychosocial aspects:

Hospital physician: "*...the patient's social situation... his or her job and things like that, things that the hospital is not geared to handle.*"

This observation corresponds with Habermas' binary codes regarding the hospital's inclusion criteria: cancer vs. no cancer (treatment – no treatment).

The psychosocial aspects, however, are the GP's primary focus, besides the biomedical obligations.

GP: "*... it is not so much the physical, we more treat them as a psychological rehabilitation. The person's integrity. That is to get back to normal ... in the family or later in a larger social and professional and vocational context.*"

Looking at Figure 1, the circle symbolizes the CSP's and the CSP's "lifeworld", the remainder of the figure around the circle illustrates the "system". The surrounding actors, written in bold, are the most relevant actors at the "system" level. One could say that the hospital makes sure that the CSP survives (or that the circle exists). The GP's primary focus, on the other hand, is to make the CSP function in the world comprised by the circle as well as "the system", sometimes with assistance from " the system" itself. This appears to be a good distribution of tasks, and it could be a good basis for a division of labour between the two groups of physicians. However, our empirical findings show that this distribution is, in fact, not working.

### The current distribution of rehabilitation between hospital physicians and GPs

While the hospital physicians assume responsibility for the treatment of the cancer, the GPs would prefer to cater for the psychosocial aspects stemming from both the cancer and its treatment. However, the organizational pathways do not facilitate this role division.

Female CSP: "*Well, I experienced, anyway, that it is like there is no connection. That is, it is well-connected ... inside the hospital ... But I just don't think that the hospital is connected to the general practitioner*"

Hospital physician: "*...we very much focus on delivering this treatment. And when you have delivered the treatment, the patient, actually, is, indeed, very much alone (...) it might be there that the primary healthcare sector could take over, but there really are no established contacts.*"

GP: "*You can say that they are on their own if they do not contact me themselves. That goes for all of them. Nothing or nobody tells them to contact me and get on with their lives. So they are left to themselves if they do not take the initiative themselves.*"

As a CSP said: "*In this respect, I think that they (the hospitals ed.) let you go too early. And somehow looks only at the purely medical aspects. And there I need a conclusion where they say, yes, but it takes more until you are back and become – yes, a whole human being.*"

This last quote illustrates that the CSP acknowledges the distinction between treatment and rehabilitation, as introduced by GPs and hospital physicians. However, the implication of this distinction as a prerequisite for treatment at the hospital does not seem clear to the CSP at the end of treatment.

Only some of the physical and psychosocial problems have biomedical contents or roots, and it seems important to distinguish the psychosocial from the biomedical aspects. This distinction could be a tool in telling CSPs that the hospital knows how to treat the cancer by means of surgery, radiation and chemotherapy, while the GPs have a wider agenda and may be able to help them get back to a relatively normal life. This distinction could also be used to bring in other actors to complete or compliment the rehabilitation effort.

The lack of profound non-medical communication concerning rehabilitation needs with other relevant actors was also observed at the municipal level. While a few CSPs had positive experiences with municipal involvement concerning their return to work, many experienced difficulties, and some felt that they were pressured back into work by the municipality after their discharge as CSPs [5]. The hospital physicians, as illustrated by this quote, also know of this problem:

Hospital physician: "*I think it's when we give them a bill of clean health, then they become trapped. Then all the social workers think that they need to push them back to work.*"

The relevance of the biomedical code of cancer patient vs. CSP cannot be questioned in relation to decisions about further surgery, chemotherapy or radiation, but it is hardly a relevant criterion for deciding whether a CSP can resume work or whether (s)he may be able to work full time. Other types of information would therefore appear more relevant for communication with the municipalities as well as for communication with the GPs.

The biomedical discourse could explain why psychosocial problems tend not to be properly dealt with within the healthcare system; they do not fit into the biomedical discourse and often cannot be treated by means of biomedical approaches. Additionally, the biomedical discourse makes it difficult to explore, identify and communicate these needs to relevant actors outside the hospitals. This combination could leave CSPs without the knowledge of how to proceed and where to go with psychosocial problems.

During treatment and hospitalization, some of the patient's lifeworld becomes colonized by the system. This hampers the CSP's ability to distinguish his or her lifeworld from that of the system, and the CSP comes to believe that the hospital can handle lifeworld problems. The colonization also obscures the fact that the patient's inclusion into the hospital system hinges on the cancer diagnosis. The exclusion of the patient's lifeworld problems from the professional dialogue within the hospital context is problematic because most CSPs fail to recognize this situation and they moreover find it difficult to introduce lifeworld problems to their GP [11]. In combination with poor conceptualization of rehabilitation needs and poor instruction on where to seek help, the CSPs are practically left in limbo at discharge, although the GPs expressly state that they do want to address non-biomedical issues in the wake of cancer.

### The general practitioner's specific knowledge of cancer

In spite of a claimed biomedical and psychosocial focus among the GPs, they tend to return to the biomedical discourse when the subject is "their opportunities for helping the CSPs". It seems that they focus on the biomedical aspects and fail to see the common traits, which are often of a psychosocial nature, that are relevant for all types of cancer. Blinded by this narrowing of their focus through the biomedical discourse, the GPs state that they do not feel capable of keeping up with the state of art in cancer treatment:

GP: "*I think that it is difficult to keep abreast of current developments, and now they have introduced a new treatment strategy for it. Actually, it seems to me that nothing is common for all cancer patients*"

The GPs' problems with keeping up to date in cancer treatment are also acknowledged by the hospital physicians:

Hospital physician: "*You can say that it is a difficult burden that is added to the GP's job, because he has to cover the whole spectrum of cancer patients ... How should he know the course (of the disease ed.).*"

Cancer and cancer treatment have obtained a nearly hegemonic status because it fits perfectly into the medical discourse. However, this status appears to be an obstacle for detecting and handling the psychosocial problems that do not depend on the continued presence of the cancer and/or its treatment, but merely originate from the original disease and treatment. It also seems that the specific type of cancer and the type of treatment applied for a specific cancer can receive a hegemonic status counterproductive to examining cancer as a common group of diseases and where CSPs share problems and treatment concerns. This hegemonic status is, however, not really relevant in relation to the rehabilitation efforts, even if the cancer and its treatment involves specific rehabilitation needs, since the rehabilitation process should be able to explore, identify and communicate all relevant rehabilitation needs.

The CSPs share the opinions voiced by the GPs and the hospital physicians in relation to the GPs' knowledge on cancer:

Male CSP: "*I don't think that the GP has the necessary knowledge. My GP did not know much about cancer, and definitely not my cancer type.*"

While the hospital physicians state that they focus on treatment and physical function, the GPs describe their focus as being mainly on the psychosocial aspects, but this division of tasks does not seem to be recognized, experienced or acknowledged by the CSPs:

Female CSP: "*But they (the GPs ed.) are probably good at their specialty, but they might be less good at involving others who are also part of it: the psychologist, the physiotherapist and the social worker. You probably have to be a little more aware of those aspects yourself.*"

This quote is in sharp contrast to the statement from her personal GP:

GP: "*Well, we actually have tools in all three areas: the biomedical, the psychological, and the social. Really, because we know the local area...or we can get help for palliative purposes... or we can refer the patient to some places... we get an idea of the presence of local self help groups which may be just the right thing for some patients.*"

Some CSPs do not think that the GP has the necessary knowledge; other CSPs simply do not think of their GP as part of their rehabilitation process. It is, however, interesting that the biomedical aspects are constantly in focus while psychosocial aspects are more briefly mentioned – and not a prominent aspect in relation to the GPs. This is probably due to the physicians' education which is, by its very nature, related to the biomedical discourse. These aspects are therefore not easily identifiable and communicable. The GPs tend to fall back into the biomedical discourse, and the hospital physicians do not pave the way for CSPs to approach the GP and take advantage of the GP's possibilities for providing psychosocial support. In this way, it becomes difficult for the CSP and the healthcare system to benefit from GPs' and hospital physicians' different foci.

### Time and accessibility

#### The CSPs' need for contact and information

As stated elsewhere, one of the most important aspects for CSPs, and also very pertinent to their rehabilitation, is their need for contact and information [5,16].

Often, but not always, the hospital ward tells the CSP to contact the hospital again if they have problems after discharge:

Hospital physician:"* We have a policy of open doors. In principle, they attend controls at specific intervals, but we also tell them that if there is anything they think is related to their illness, then they can come here... They can just call, and then they can get an appointment on very short notice.*"

Female CSP: "*... They told me, just call if there is anything, even if it is in the middle of the night, call the hospital ward. They know you and know how and why. You should not be worried about something at home.*"

This quote shows, as mentioned by other CSPs, that they think that the hospital ward knows the CSPs and the CSPs' conditions. This is probably a product of the colonization by the hospital system, and it is reinforced by the policy of the open doors. However, the quote of the hospital physician is mainly linked to the biomedical discourse of cancer treatment and does not include any psychosocial aspects.

### Insufficient rehabilitation opportunities

As presented earlier, the possibility of contact and information is important to the CSPs, but this is also an important aspect for GPs and hospital physicians in relation to rehabilitation. Hospital physicians sometimes have trouble finding information on the rehabilitation possibilities offered by society.

Hospital physician: "*Sometimes, I doubt that we really grasp the rehabilitation possibilities that society offers, and we don't know where to learn more about new offers. I think that can be a problem.*"

The frustration among hospital physicians concerning the organization of rehabilitation, as well as the information on available possibilities, is illustrated by this quote from a local hospital physician:

Hospital physician: "*...We don't have any proper rehabilitation in this country. Not for the types of cancer I know of, anyway. We do in relation to the purely surgical issues – like, right afterwards. And we check up on the wound. We check up on a stiff shoulder and things like that. But all the other things like getting back to life, getting back on the job, getting back to the social life and sexual life for that matter and things like that, there we have nothing!*"

As illustrated by the last quote, the oncologists are focused on the treatment and the physical rehabilitation needs related to the treatment, but physicians from local hospitals mentioned in particular that they lacked offers to the CSP, and they all wanted to see a proper organization of the rehabilitation efforts. While they were aware of other possible rehabilitation needs, it is clear from the quotes above that this was not their main priority.

#### GPs request more information from the hospitals

The GPs do not know the CSPs' situation and needs after discharge, and this makes it difficult for them to take active part in the rehabilitation effort. Such information would in all likelihood make it easier for the GPs to help the CSPs.

GP: "*I think I know too little. To be able to talk properly with the patient. So, I feel my way. What has she been told, or more correctly, what does she think she has been told. Because it's not certain, it was exactly what was said that she understood. It is often something quite different.*"

There are no established structures for passing on such information to the GP or other relevant actors, despite the fact that the hospital physicians are perceived to have the best knowledge of the CSPs' rehabilitation needs at discharge. The problem may be that the psychosocial rehabilitation needs do not fit into the biomedical discourse, which may make it difficult to assess and communicate these needs within and across these subsystems.

### The CSPs' rehabilitation needs

Another aspect that emerged, is the problem of assessing the CSPs' rehabilitation needs; this aspect was mainly mentioned by the hospital physicians, and, again, the focus was on the physical aspects, which was is to be expected when evaluating a medical treatment:

Hospital physician:" *We interview on side effects after surgery... " Quoting the patient: "So we (the CSPs ed.) will, of course, not tell the nice physician, who cured them, that it is actually not that well*".

While the hospital physicians are aware of the fact that the psychosocial aspects do exist and are relevant to CSPs, they do not attempt to access them systematically, and they doubt that they know all about the CSPs' needs:

Hospital physician: "*How well do we know Mr. Jensen? What is the size of the garden he used to take care of? And well, now he says he is not able to do it any more*"

This quote still operates within the precinct of the biomedical discourse – the aspect of Jensen's garden is not a biomedical aspect and is therefore not investigated.

Despite the fact that the hospital physicians are perceived to be the professionals with the most profound knowledge of the CSPs' possible needs at discharge, they find it difficult to assess the CSPs' rehabilitation needs. When the assessment is done, they primarily assess the physical deficits stemming from cancer and treatment. Once again, this fits nicely into the biomedical discourse, and it seems to explain why the psychosocial aspects are not assessed. Involvement of other actors or another part of the healthcare sector, e.g. the municipalities or the GP would otherwise appear natural, but it cannot be accomplished within the biomedical discourse, and that is probably the principal problem for rehabilitation in general and for psychosocial rehabilitation in particular.

### How could cancer rehabilitation be organized?

As shown above, cancer rehabilitation is poorly organized. This goes for both physical and psychosocial rehabilitation needs, which are, at best, randomly assessed by the physicians. It is probably because of a lack of understanding, among both physicians and CSPs, of the fundamental difference between the system and the lifeworld and a lack of ability among professionals to operate within the realm of a non-biomedical discourse. This lack of ability seems to be a product of the biomedical discourse focusing on the biomedical aspects; while the hospital physician in the last quote recognizes that other aspects are relevant to the CSPs, they are not relevant in the biomedical discourse.

If the CSPs were referred to the GP for additional control in relation to the cancer, it would both satisfy the biomedical discourse and give the GP a chance to introduce additional psychosocial discourse. This can be illustrated by the following quote:

GP:"* This is an ongoing process. Even if they (the CSPs ed.) experience that it ends. It's something that has to continue... But they (the hospital physicians ed.) need to be a little better in telling that life goes on.*"

The GPs plan from a long-term perspective and ask for a level of involvement that seems realistic:

Hospital physician:"*... even if we are not implementing it, then it should be mentioned in the electronic case note. That there are these needs and that something must be done about them. It is really important, since, otherwise, it will not be taken care of.*"

GP: "*I think that the GP should be a kind of anchor person for the patient.*"

The GPs and the hospital physicians share the impression that the assessment of the CSPs' psychosocial rehabilitation needs is unsatisfactory, and that there is insufficient follow-up of the CSPs' needs after discharge. The open question is whether the hospital physicians could do more to encourage the CSPs to visit the GP and his more lifeworld-orientated discourse. Whether the GPs really would do better at assessing these needs has, to our knowledge, not been shown. However, the quotes above illustrate that a structured process for assessing former cancer CSPs' rehabilitation needs may improve our knowledge of the CSPs' actual needs.

### Improved assessment and referral process

The problems, as outlined above, could be dealt with through enhanced assessment of rehabilitation needs and by an improved discharge process:

Hospital physician: "*Maybe, a good discharge note should contain some completely different aspects, something about expected working capabilities after how long and such things.*"

Hospital physician: "*This quality of life can probably also be achieved if a good handover procedure is established between the hospital and the GP. And the patient has complete confidence in the GP's control of what will happen onwards.*"

GP: "*... a model could be that you had this discussion with the patient at discharge. What do you feel your needs are? And then started from that. Someone could say, yes, I would like to follow up on this with my GP in a fortnight,... but it is an offer that the patient can take advantage of at discharge.*"

Hospital physician: "* A form that the physician fills in at discharge. Dealing with physical and psychological aspects. The social aspects and a whole lot of other things. It could be a questionnaire you are forced to go over ... together with the patient. You can say that that may provide a better basis than the two lines of text in the discharge note, currently being written. Where it just says that the patient has finished chemotherapy.*"

Male CSP: "*...these aspects should either be discussed at the discharge consultation. Making some kind of rehabilitation plan. Or it should be done by the GP. However, (...) I don't think that the GP has the required knowledge.*"

The GPs, like the hospital physicians, were open for some sort of checklist or guide for the GPs, exploring aspects that could be relevant to the CSPs' needs. While the hospital physicians appear very open to this idea, the GPs are also positive, but they would prefer to use a checklist as guidance on possibly relevant aspects rather than a rigid checklist.

### Who would the CSPs prefer as their anchor person?

It has been reported elsewhere that CSPs often feel left in limbo after discharge [5,17,18].

While CSPs, GPs and hospital physicians agree on how to arrange a good discharge in relation to the rehabilitation process, many former cancer CSPs did not assign the GP an active role. This could partly be explained by the quote above from the 52-year-old male stating that he did not expect the GP to have the required knowledge. Instead, the CSPs felt – probably because of the colonization process – that the hospitals had the required knowledge as well as interdisciplinary resources:

Female CSP:"*... it could be nice to have a nurse, someone you had had as a contact person or talked to at the hospital, that it was her who called you (after discharge ed.)*"

Another female CSP: "*I think that it can work as it does today if it was possible to use the interdisciplinary resources which actually exist within the hospital. They have a dietician, and there is physiotherapy, too, and there is a psychologist and a hospital priest. (...) Attach a network of volunteers who have had the disease themselves. And then some information possibilities, for example on the Internet.*"

The two CSP quotes above assign the role as "anchor person" for the rehabilitation process to the hospitals. In the first quote, the CSP emphasizes that the she would prefer that the tailoring of the rehabilitation after discharge was done by someone knowing the CSP really well – a hospital nurse. This CSP had been in treatment for a long time and stated that her social network had shrunk during and after the treatment period. This could indicate what Habermas calls colonization of the lifeworld where the CSP's lifeworld has shrunk while the personnel from the system of the hospital have come to play a larger role in the CSP's life.

The two quotes above also underline that different types of professionals may be brought into the rehabilitation process. Even though the CSPs prefer these professionals to be working inside the hospital setting, this may not be necessary as long as the needs are systematically assessed by a hospital physician or nurse who assesses biomedical and psychosocial rehabilitation needs. This is supported by the fact that the CSPs who assigned the GP a larger role in the rehabilitation process were those who had been treated several years ago and had congruent diseases and/or CSPs who were not given adjuvant therapy. These CSPs may be expected to experience a lesser degree of colonization by the hospital services and they are therefore more prone to seek out the GP:

Female CSP: "*I would like the GP to be committed to contacting the patient within one week after discharge from the hospital.*"

This is also supported by the following quote from a GP:

GP: "*There is a big difference depending on where people are discharged from. If they are discharged from the radiation therapy clinic or from surgery, for example. After an operation there is not really any follow-up. I think that they are more in need of knowing that they can come to us for talks and follow-up than those who come from the radiation therapy clinic.*"

Based on the above mentioned idea of an extended discharge consultation at the hospital, both GPs and hospital physicians proposed that during the discharge consultation at the hospital CSPs should be encouraged to establish contact with their GP shortly after discharge. In addition, most of the CSPs wanted the hospital physicians to assess their rehabilitation needs. Such an arrangement, as also proposed by the hospital physicians and the GPs, is likely to bring the CSPs out of the unintended colonization and could bring them closer to their social network and the other resources available in the lifeworld.

## Discussion

Our analysis, theoretically inspired by Habermas, showed that the biomedical discourse imbedded in the system world promotes the strategic actions related to the disease and does not capture the psychosocial aspects of cancer. In addition, the CSP's lifeworld often becomes colonized by the system during hospitalization, making discharge from the hospital difficult. These two aspects and the theoretical understanding of the process seem crucial to our understanding of why CSPs, as reported elsewhere, often feel left in limbo after discharge [5,17,18].

The focus of this study was the organization of cancer rehabilitation. This issue was assessed through FGIs with GPs, CSPs and hospital physicians as well as through individual interviews with GPs. This does not provide 'objective' data of how the discharge process and cooperation works, or of how many are rehabilitated and in which way, but it does provide insight into the experiences of these three groups with the rehabilitation of CSPs.

Earlier studies have shown organizational deficits in cancer care concerning the cooperation between GPs and hospital physicians, especially with regard to the failure to meet CSPs' psychosocial needs [19,20]. If the GPs are to assume a more important role in the aftermath of cancer therapy, the hospitals would have to provide them with more information upon discharge [21]. The theoretical basis presented in this study could be important in improving the discharge process since it furthers an understanding of the processes going on during treatment and hospital stay.

Physicians should be aware that the biomedical discourse addressing the strategic aims of cancer treatment obscures a broader discourse that also comprises psychosocial aspects. The hospital physicians are focused on the biomedical cancer treatment; the GPs as well as the hospital physicians think that the GPs would be well-suited to take over after discharge from the hospital and take care of biomedical as well as psychosocial aspects. Nevertheless, the impact of this task distribution and the impact of the different discourses (communicative vs. strategic actions) do not seem clear to the staff or the CSPs involved, and there is often no active cooperation between different sectors within the healthcare system. There is currently no formalized contact or set-up ensuring proper communication and a careful handover of CSPs upon discharge. Currently, the discharge note from the hospital contains the treatment status of the CSP and the principal biomedical code for inclusion or exclusion into the hospital: cancer/not cancer. No information is given on psychosocial rehabilitation needs. This format fits very well into the strategic actions of the biomedical discourse – but not into the CSP's life. This may be due to a lack of understanding of the meaning of system and life world and of the importance of the different discourses. These dimensions could be important tools for improving the rehabilitation process.

The use of the biomedical discourse at discharge seems to lie at the root of the difficulties of communicating psychosocial aspects between the subsystem of the hospital physicians and that of the GPs. An alternative would be to combine this discourse with the discourse of health, very similar to the WHO definition of rehabilitation used in this study, which can be defined as:

"*Health may therefore be defined as "the state of optimum capacity of an individual for the effective performance of the roles and tasks for which he has been socialized. It is thus defined with reference to the individual's participation in the social system*" [22].

In other words, healthcare personnel have to remember that while the biomedical discourse focuses on strategic actions, like understanding which disease the CSP has, as a precondition for treatment [23], the rehabilitation aims rather at understanding the CSP who had the disease (achievable through communicative actions).

Seen in this light, the biomedical discourse appears to pose an obstacle to proactive rehabilitation.

This problem appears to be amplified by the (always!) unintended colonization of the CSP's lifeworld during hospitalization. This colonization may reduce the CSP's feeling of social belonging and may disturb the CSP's identity [10]. Moreover, the colonization of the CSP's lifeworld can make the CSP unable to distinguish between the system at the hospital, merely as a service aimed at curing cancer, and the lifeworld constituted by the CSP's own social network. Consequently, the CSPs feel left in limbo after discharge, longing for their "new" "pseudo" network at the hospital and often failing, or at least finding it difficult, to seek other rehabilitation services outside the hospitals and closer to their lifeworld.

Other studies have shown that patients may find it difficult to introduce lifeworld aspects during a consultation with the GP [11]. Such experiences may make the CSPs reluctant to consult the GP about psychosocial problems after discharge. This reluctance may be amplified if the CSPs have just been discharged from the hospital, which, though situated within the biomedical discourse, was perceived to constitute an important part of their lifeworld. In this way, the biomedical discourse could "fool" the CSP into dismissing the GP as a relevant partner in the rehabilitation process after discharge from the hospital.

At the same time, the GP seems to draw mostly on the biomedical discourse when he deals with difficult and complicated problems, such as those of CSPs. This is to be expected since the biomedical discourse is the backbone of the medical education, but our results show a need for promotion of the psychosocial part of the GP's working area since the GPs state that they find this part of their work important and a natural part of being a GP.

An strengthened rehabilitation process may be achieved if the hospital physicians become aware of these aspects and help the CSPs to find their way back into settings outside the hospital, closer to their lifeworld. One way could be to refer the CSP to the GP and to combine this with improved communication, extending the biomedical discourse. Concurrently, GPs should be aware of the dualism between the biomedical discourse and the health discourse. This knowledge could be a helpful tool, enabling the physicians to be proactive in inviting the CSPs to seek help from the primary care sector in their rehabilitation process.

We chose to mainly focus on the physicians' performance in this study. As seen from some of the quotes, many other professions, e.g. nurses, physiotherapists and social workers are also involved in the rehabilitation process. It would be interesting to conduct an analysis of the dynamics among these professions in relation to the system and the lifeworld and varying professional discourses. It would also be interesting to investigate the possible influence of the biomedical discourse on the performance of these groups. Finally, it is interesting if the new organisation of the rehabilitation where the Danish municipalities now are responsible for rehabilitation will be a barrier or foster a fruitful organization of the rehabilitation based on cooperation between hospitals, GPs and municipalities.

## Conclusion

In relation to CSPs, an improved discharge process may prove helpful, since the GPs want to know more about the information given to the CSP and about the CSP's physical and psychosocial condition. Part of this information could be made accessible to and understandable for, e.g. social workers. In addition, the CSP could be referred back to the GP at discharge, ensuring that the necessary rehabilitation possibilities are offered. Finally, it is necessary to gather information on existing rehabilitation possibilities and make the knowledge of these possibilities accessible to CSPs, GPs and hospital physicians.

Our analysis shows that the success of formal processes, checklists and enhanced discharge processes depends on the involved healthcare personnel's theoretical and practical awareness and knowledge of the distinction between system and lifeworld and the possible colonization of the CSP's lifeworld during treatment. In addition, it is important to be aware of the limitations of strategic actions in the biomedical discourse in relation to determining and dealing with different types of rehabilitation needs. Without this knowledge, the suggested enhancements are likely to fail.

## Abbreviations

GP: General practitioner; CSP: Cancer surviving patient.

## Competing interests

The authors declare that they have no competing interests.

## Authors' contributions

This study was conceptualized and drafted by THM, JS, FO and ABJ. THM, JS and FO conducted the FGIs. THM and JS drafted the manuscript and FO and ABJ revised the manuscript critically. All authors read and approved the final manuscript.

## Ethical approval

The study was approved by the Danish Data Protection Agency (j.no. 2006-41-6468) and recommended by the Multi-Practice Committee of the Danish Society of General Practitioners and The Organisation of General Practitioners in Denmark (MPU 6-2006). According to the Scientific Committee for the County of Aarhus, the Biomedical Research Ethics Committee System Act does not apply to this project.

## Pre-publication history

The pre-publication history for this paper can be accessed here:


